# The Role of the Microbiome in the Pathogenesis and Treatment of Asthma

**DOI:** 10.3390/biomedicines11061618

**Published:** 2023-06-02

**Authors:** Katarzyna Logoń, Gabriela Świrkosz, Monika Nowak, Martyna Wrześniewska, Aleksandra Szczygieł, Krzysztof Gomułka

**Affiliations:** 1 Student Scientific Group of Adult Allergology, Wroclaw Medical University, 50-369 Wrocław, Poland; katarzyna.logon@student.umw.edu.pl (K.L.); gabriela.swirkosz@student.umw.edu.pl (G.Ś.); martyna.wrzesniewska@student.umw.edu.pl (M.W.); aleksandra.szczygiel@student.umw.edu.pl (A.S.); 2 Clinical Department of Internal Medicine, Pneumology and Allergology, Wroclaw Medical University, 50-369 Wrocław, Poland; krzysztof.gomulka@umed.wroc.pl

**Keywords:** asthma, microbiome, gut microbiome, bronchial microbiome, prebiotics, probiotics

## Abstract

The role of the microbiome in the pathogenesis and treatment of asthma is significant. The purpose of this article is to show the interplay between asthma and the microbiome, and main areas that require further research are also highlighted. The literature search was conducted using the PubMed database. After a screening process of studies published before May 2023, a total of 128 articles were selected in our paper. The pre-treatment bronchial microbiome in asthmatic patients plays a role in their responsiveness to treatment. Gut microbiota and its dysbiosis can contribute to immune system modulation and the development of asthma. The association between the microbiome and asthma is complex. Further research is necessary to clarify which factors might moderate that relationship. An appropriate gut microbiome and its intestinal metabolites are a protective factor for asthma development. Prebiotics and certain dietary strategies may have a prophylactic or therapeutic effect, but more research is needed to establish final conclusions. Although the evidence regarding probiotics is ambiguous, and most meta-analyses do not support the use of probiotic intake to reduce asthma, several of the most recent studies have provided promising effects. Further studies should focus on the investigation of specific strains and the examination of their mechanistic and genetic aspects.

## 1. Introduction

Asthma is a chronic inflammatory disorder of the airways and is one of the most common non-communicable diseases, affecting more than 300 million people worldwide. It is characterized by airflow obstruction, bronchial hyperresponsiveness, change in mucus production, remodeling of the airway wall, and symptoms such as wheezing, coughing, chest tightness, and shortness of breath. The prevalence and severity differ globally, with asthma being more common in boys in childhood and affecting more women than men in adulthood [1,2,3]. The pathophysiology of asthma is complex and involves interactions between immunological, genetic, and environmental factors. Chronic inflammation of the airway wall, mediated by various immune cells such as dendritic cells, eosinophils, neutrophils, lymphocytes, innate lymphoid cells, and mast cells, leads to airway obstruction. Two phenotypes of asthma can be distinguished: eosinophilic and neutrophilic. In eosinophilic asthma, Th2 cells or ILC2s that produce cytokines such as IL-4, IL-5, and IL-13 mediate inflammation, promoting hallmark features of asthma such as eosinophilia, mucus hypersecretion, bronchial hyperresponsiveness, and IgE production. Neutrophilic asthma is characterized by neutrophilic infiltration and secretion of interferon gamma and IL-17 by Th1 and Th17 cells [2,4,5]. There is a considerably higher rate of prevalence of comorbid conditions such as obstructive sleep apnea, nasal polyposis, gastroesophageal reflux disease, allergic rhinitis, obesity, depression, diabetes mellitus, and cardiovascular diseases among asthmatic patients, especially in those with severe asthma [6]. Treatment of asthma aims to achieve and maintain control of symptoms, reduce the risk of exacerbations, and improve quality of life, and includes both pharmacological and non-pharmacological interventions such as inhaled corticosteroids, bronchodilators, immunomodulators, patient education, exercise, smoking cessation, and rehabilitation [7].

The human microbiome is a highly diverse and complex community of microbes that include bacteria, viruses, fungi, and protozoa, as well as their genes and metabolic products that colonize all body sites such as the oral cavity, skin surface, gastrointestinal tract, esophagus, and lung. The microbiome is shaped and modified by various genetic, dietary, and environmental factors—mode of birth, age, and antibiotic intake [8]. Functions of the microbiome include modulation of the immune system, supporting digestion and metabolic processes, modifying insulin secretion, and cells’ insulin resistance. The relationship between the microbiome and the host’s organism is mainly mutualistic. Changes in the composition and function of the human microbiome affect intestinal permeability, digestion processes, body metabolism, and immune responses [9]. Metabolites produced by gut microbiota function as signaling molecules that transmit information to different body organs, affecting the immune system. These synergistic connections are described as microbiota–gut–lung, microbiota–gut–skin, microbiota–gut–oral, and microbiota–gut–brain axes [10,11]. By gut–organ axis communication and the microbiota’s impact on immunological processes, alterations in gut microbiota may have a significant impact on the development of diseases of the lung, skin, brain and other organs and systems, including neurodegenerative diseases [12], dementia, cognitive frailty [13], and cardiovascular diseases [14]. A dysbiosis of the lung microbiome is observed in asthma and is likely a cause of asthma manifestation. Some bacterial metabolites, especially short chain fatty acids, have protective properties in airway inflammation. One hypothesis regarding the development of asthma suggests that immune system dysfunction results from a lack of exposure in infancy to a variety of environmental microorganisms [10,15].

In this review we wanted to summarize recent studies on the subject of the human microbiome and its role in asthma pathogenesis and treatment and to highlight the main areas that require further research.

## 2. Human Microbiome

The human microbiome is the aggregate of all microorganisms (the collection of microbial genomes) that reside on or within human tissues and biofluids. The gut is the main location of the human microbiome, but microbiota can reside also on the skin and in mammary glands, seminal fluid, the uterus, ovarian follicles, the lung, saliva, the oral mucosa, the conjunctiva, and the biliary tract [16]. The total number of bacteria in the human body is around 3.8 × 10^13^ [17].

The gastrointestinal microbiome is a diverse consortium of bacteria, archaea, fungi, protozoa, and viruses that inhabit the gut of all mammals. Before birth, humans are sterile, with inoculation with microbes occurring at the time of birth [18]. At birth, the microbiota is aerobic, with low numbers and low diversity, with the most common bacteria being facultative anaerobes and members of the Enterobacteriaceae phylum [19]. Some studies suggest that the early colonizers of the infant intestine are acquired through contact with maternal microbes [20]. Newborns delivered by cesarean section acquire different microbiota to infants delivered vaginally [21]. Some studies showed that the gut microbiota of cesarean section newborns contains higher numbers of bacteria such as *Clostridium perfringens* or *Escherichia coli*, which are potentially pathogenic, and lower numbers of the common gut microbiome genera, such as *Bifidobacterium*, *Streptococcus*, and *Lactobacillus*, compared with vaginally delivered newborns [22,23]. In promoting a healthy microbiota in neonates, the following are also important: delivery at term, breastfeeding, and exposure to a variety of microorganisms [24,25]. With the introduction of solid food, a more adult-like microbiome starts to develop as of 6 months of life [24].

The human colon ecosystem contains more than 400 bacterial species [26]. An adult’s gut appears to have a unique microbial community but the anaerobic genera *Bacteroides*, *Eubacterium*, *Peptococcus*, *Peptostreptococcus*, *Clostridium*, *Ruminococcus*, and *Faecalibacterium* have typically been found [20,26]. Archaea, which is another large class of gut flora, are important in the metabolism of the bacterial products of fermentation. Genera such as *Escherichia* and *Lactobacillus* are present to a lesser extent [27]. Fungal genera in the gastrointestinal microbiome are, for instance: *Candida*, *Saccharomyces*, *Aspergillus*, *Penicillium*, *Bullera*, *Pleospora*, *Rhodotorula*, *Trametes*, and *Galactomyces* [28]. *Bacteriophages* are the most common virus genera [29].

The gut microbiota has impacts including resistance to pathogens, maintaining the intestinal epithelium (for example, commensal bacterial species such as Lactobacillus stimulate Toll-like receptor 2), metabolizing dietary and pharmaceutical compounds, and controlling immune function [24,30]. Immune surveillance and defense are provided by the gut-associated lymphoid tissue (GALT). GALT is a component of the mucosa-associated lymphoid tissue (MALT) which works in the immune system. GALT must sample luminal bacteria and other antigens to evoke immune responses against them. Lymphoid follicles containing immune cells are poised to initiate either an inflammatory or an anti-inflammatory response depending on the specific microbial signals. M cells (where M stands for microfold or membranous), which are part of GALT, are a unique intestinal epithelial cell (IEC) subset that is responsible for the immune sensing of luminal bacteria [31]. Moreover, research carried out by Stepankova et al. showed that microbes are necessary in the development of GALT, as evidenced by severely underdeveloped lymphoid tissues in germ-free animals [32].

There is a clear difference in dynamics and quantity between the lung microbiome and other human microbiomes [33]. In the past, researchers thought that healthy lungs were sterile, but studies using rigorous sampling revealed that bacterial sequences are usually detectable in the lung. Proximal lung regions share greater bacterial levels than bronchioles [33,34]. The most common genera in the respiratory system are *Prevotella*, *Streptococcus*, and *Veillonella*, while *Firmicutes* and *Bacteroidetes* are the most common phyla [34]. The upper respiratory tract (URT) has a high microbial biomass while that in the lungs are low, which suggest that the lung microbiome is largely transient and composed of URT-derived immigrants. Lungs are cleared from the URT microbiome through mucociliary and innate immune mechanisms [35].

Fungal communities (mycobiome) of the lung are understudied. It is still unknown if the lung mycobiome contributes to normal physiology. Nevertheless, *Ascomycota*, *Basidiomycota*, *Candida*, *Penicillium*, *Saccharomyces*, *Cladosporium*, and *Fusarium* are the most identified genera and taxa [33,36] (Table 1).

## 3. The Molecular Role of the Gut Microbiota in Asthma Pathogenesis

**Figure 1:**
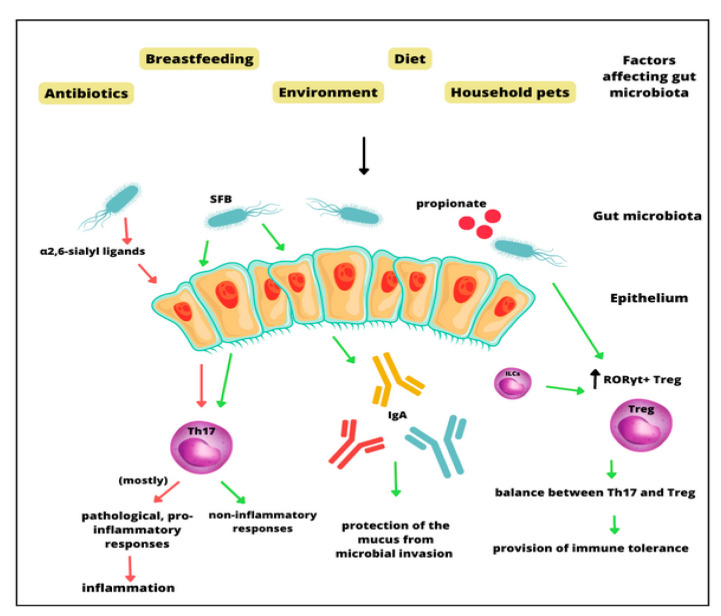
Interactions between the gut microbiota and immune reactions.

Understanding interactions between intestinal microbiota, host, and asthma pathophysiology is crucial to determining immunological mechanisms. Interactions between host immune cells and the gut microbiota are important for the development of the immune system and homeostasis in the respiratory tract. Microbial interaction with the immune system is crucial in maintaining proper immune responses which prevent the development of both infectious and non-communicable diseases. During research conducted on germ-free mice, it was revealed that the gut adaptive immune system is suppressed in these animals. What is more, the introduction of commensal bacteria to their microbiota could induce the development of mucosal CD4+ T cells and cytotoxic CD8+ T cells [37]. T helper 17 cells (Th17), which play an important role in host protection as well as in inflammatory responses, can support pathogenic effector T cells in extra-intestinal diseases. Specific microbes, such as segmented filamentous bacteria (SFB) and other commensal species, can induce such responses. Additionally, Th17 responses can also be influenced by the gut microbiota [38]. The microbiome-dependent Th17 inflammation is regulated by α2,6-sialyl ligands; α2,6-sialyltransferase deficiency can induce mucosal Th17 responses [39]. Actinobacterium *Eggerthella lenta* can also promote pathological Th17 cells through the cardiac glycoside reductase 2 enzyme. Other immune cells whose activity is modulated by the gut microbiota are Treg cells—exposure to microorganisms as well as diet can induce Treg production [38]. This process happens via several mechanisms. Microbiota-specific RORγt+ Treg cells can be selected by innate lymphoid cells, which prevents the expansion of Th17 cells and causes intestinal immune tolerance [40]. On the other hand, some species such as *Helicobacter* spp. and *Akkermansia muciniphila* can induce RORγt+ Treg cell-mediated immune responses. A microbial metabolite, propionate, in lowered levels might cause pathological differentiation of Th17/Treg cells [41]. Another part of the immune system that gut microbiota contributes to is IgA production. It has been documented that germ-free animals have smaller Peyer’s patches and that intestinal IgA levels are severely induced in these organisms [42]. It is also suggested that specific microbes can induce intestinal IgA more significantly; for example, colonization with segmented filamentous bacteria (SFB)–mouse symbiotic organisms causes an increase in intestinal IgA production. What is more, mice with intestinal SFB colonization have higher intestinal IgA levels than those colonized with *Clostridium* (Figure 1).

Allergic reactions can be altered by influencing the receptors of innate immunity, Pattern-Recognizing Receptors (PRRs), among which the most studied are Toll-like Receptors (TLR), Nod-like receptors (NLRs), C-type lectin receptors (CLR), retinoic acid-inducible gene-I (RIG-I)-like receptors (RLRs), and nucleotide-binding domain receptors [43]. It is known that commensal bacteria in the gastrointestinal tract have the ability to regulate immune responses in both local and extraintestinal mucosal defenses. PRRs of the intestinal mucosa are influenced by microbiota, which causes maturation of the intestinal immune system, local production of antimicrobial peptides, and killing of intestinal pathogens [44,45]. Microbiota depletion can cause significant deterioration of the innate response mechanisms (especially reduced reactive oxygen-mediated bacteria killing by macrophages); on the other hand, administering bacterial ligands of NLR can rescue host lung defense mechanisms [44]. What is more, what rescues immune defense mechanisms in the lung are gastrointestinal, not upper respiratory, NLR ligands.

It has been found that bacterial compounds—lipopolysaccharides (LPS) and muramyl peptides (MDP)—formed by the natural decay of microflora interact with the receptors of innate immunity and result in immune reactions to get rid of the pathogen. LPS are agonists of TLR receptors and MDP are NLR ligands that react with innate receptors on the bronchial epithelium and immunocompetent cells; these molecules are small particles of the bacterial cell wall which cause immunological reactions such as the release of pro-inflammatory cytokines and mediators, but they can also control the intensity of the inflammatory process [46]. Lipopolysaccharides, through interacting with the TLR4 and muramyl peptides, trigger antibacterial protection through NOD2 receptors. Dysregulation between these interactions can cause chronic inflammation, asthma, or autoimmune and allergic reactions. In an experiment conducted on mice, inducing airway inflammation was either preceded by an intraperitoneal injection of GMDP or LPS, or the innate receptor agonists were injected at the same time as the allergen. It has been found that prolonged stimulation with low doses of an innate immunity receptor agonist before sensitization by an allergen reduces the severity of the allergic process. In mice, multiple administrations of LPS and GMDP before sensitization reduced inflammation. In this group, lower serum levels of IgA and IgE have been noted. Joint administration of LPS or GMDP together with an allergen significantly increased inflammation (expressed in neutrophilia and eosinophilia) and increased serum levels of IgA and IgE. 

Bacterial metabolites, such as short-chain fatty acids, secondary bile acids, lactic acid, and bacteriocins, have antimicrobial activities which give them the ability to protect against pathogenic bacteria [47]. Commensal microbes produce short-chain fatty acids (SCFAs) which maintain immunological homeostasis, mostly by DAC inhibition, promotion of IgA production, GPR signaling, and inhibition of pro-inflammatory cytokine secretion. SCFAs are produced by the fermentation of indigestible carbohydrates by some species, including *Faecalibacterium prausnitzii*, *Roseburia intestinalis*, and *Anaerostipes butyraticus*. Recently, research confirming the hypothesis that there is a correlation between low SCFAs in the intestines and asthma development has been conducted—it was discovered that the highest levels of butyrate and propionate found in infants’ stools were connected with a lower risk of asthma and other allergic disorders. Another study on mice showed that a high-fiber diet that caused increased SCFAs levels decreased susceptibility to allergic airway inflammation [48]. In research conducted on mice, SCFAs have been proven to increase the expression of the transcription factor FOXP3 by inhibiting histone deacetylation, which supported the expansion of Treg cells and increased production of IL-10 [15]. Acetate, produced mostly by *Bifidobacteria* spp., by activating the G-protein receptor (GPR), regulates intestinal inflammation and maintains intestinal epithelial barrier function. It also induces colonic IgA production through GPR43 signaling, and it is an essential metabolite to effectively reduce Th1/Th17 and elevate Treg levels, which, as a consequence, reduces inflammation [49]. Butyrate’s main role is to serve as a source of energy for colonocytes and to promote the release of mucin. However, it also responsible for promoting the epithelial barrier through repression of Claudin-2, inducing monocyte-to-macrophage differentiation by inhibiting histone deacetylase 3 (HDAC3), and increasing expression of IFN-γ and granzyme B in CD8+ T cells. Butyrate can induce IL-22 secretion from T cells by promoting the expression of the aryl hydrocarbon receptor (AhR) and hypoxia-inducible factor 1α, and, similarly to acetate, it has the ability to modulate immune responses by activating GPR43 and inducing differentiation of Foxp3+ CD4+ Treg cells. Butyrate also accelerates fatty acid oxidation and inhibits HDAC, which promotes Treg production [50].

**Figure 2:**
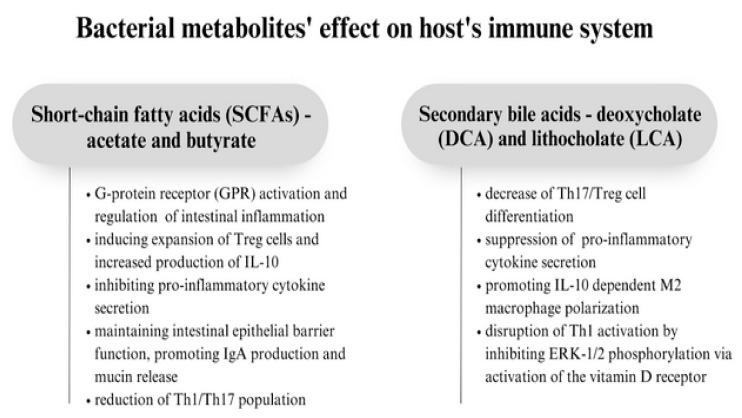
Summary of the effect of bacterial metabolites on the host’s immune system.

Secondary bile acids—deoxycholate (DCA) and lithocholate (LCA)—are products of the primary bile salt hydrolase and dehydroxylation process which takes place in the small intestine and is processed by clock-controlled intestinal bacteria. In general, they cause the decrease in Th17/Treg cell differentiation, suppress pro-inflammatory cytokine secretion, and promote M2 macrophage polarization, i.e., they modulate the gut microbiota–immune axis. Secondary bile acids activate Takeda G protein-coupled receptor 5 (TGR5), whose signaling inhibits monocyte-derived dendritic cell (DC) activation and NF-κB signaling and, consequently, promotes IL-10 dependent M2 macrophage polarization. Secondary bile acids can modulate gut RORγ+ Treg homeostasis—3β-hydroxydeoxycholic acid (isoDCA) produced by Bacteroide consortia enhanced the peripheral generation of RORγt+ Treg cells by antagonizing the nuclear Farnesoid X receptor (FXR) on DCs. On the other hand, secondary bile acids, such as 3-oxoLCA and isoLCA, suppress Th17 cell function by inhibiting their key cell-promoting transcription factor, RORγT. Unconjugated LCA, by activating the vitamin D receptor, also disrupts Th1 activation by inhibiting ERK-1/2 phosphorylation [38] (Figure 2).

Microbial dysbiosis (altered composition and function of the microbiota) in the gut, caused by lifestyle changes and environmental exposures, may result in an imbalance between species of bacteria and, therefore, lead to inflammatory system disturbances. It has been shown to contribute to allergic diseases, especially asthma pathogenesis. Several studies have reported alterations in the gut microbiota composition in asthmatic patients. It was discovered that children with an increased abundance of *Streptococcus* and *Bacteroides* and a decreased abundance of *Bifidobacterium*, *Ruminococcus gnavus*, *Akkermansia*, and *Faecalibacterium* genera have a higher risk of developing atopy, asthma, and wheezing. Another research conducted on the ovalbumin-induced mice model showed that representative species of the *Lachnospira*, *Veillonella*, *Faecalibacterium*, *and Rothia* genera can decrease airway inflammation. What is more, it was shown that there is a reduction in LPS biosynthesis pathways by gut microbiota in children with a high asthma risk [15]. Gut dysbiosis, due to the abundance of commensal bacteria, promoted opportunistic pathogen invasion which was caused by imbalanced immune responses, such as compromised gut barrier permeability; induced Th1 and Th17 cell production; excessive engagement of neutrophils with inducing pro-inflammatory cytokine release; matrix metalloproteinase production; and suppression of Treg, AMPs and IgA secretion; which, as a result, led to the encroachment of bacteria and the release of their toxic metabolites [38].

Proper functioning of the respiratory tract requires proper functioning of the airway mucosa. Pseudostratified ciliary epithelium and secretory cells provide mucociliary clearance (MCC)—an innate lung defense mechanism that allows the clearance of airways and expulsion of agents. The ciliary epithelium’s physiology can be easily affected by respiratory infections. It has been discovered that coronaviruses (including SARS-CoV-2) can cause the development of asthma [51]. After SARS-CoV-2 invasion in the respiratory tract, the virus may activate the host immune system, resulting in activated mucins (MUC5AC and MUC5B) and excessive production of mucus. The infection might also result in altering the c-AMP-dependent ciliary beat frequency, impacting host proteins controlling the intraflagellar transport machinery and ciliogenesis. This can cause cytoplasmic dynein 2 dysfunction, cilia disassembly, dysregulated ciliation, and, as a result, various ciliopathies. 

## 4. The Microbiome as a Moderator in Asthma Development

Initially proposed to elucidate the connection between microorganism exposure, decreased atopy, and allergic diseases risk, the hygiene hypothesis [52] proved insufficient in explaining the host–microorganism interplay in asthma due to its disregard of respiratory viral infections and the role of non-pathogenic microbes [53]. Subsequently, the hygiene hypothesis was adjusted, and it evolved into the “old friends’ hypothesis” [54], which highlights the role of immune response modulation after birth by specific commensal microorganisms from the microbiota in protecting the host from hypersensitivity to common antigens.

Previous research has shown that an immature microbial composition in one-year-old children is linked to an increased risk of asthma at age five, particularly among those born to asthmatic mothers and with allergic sensitization [55]. The findings suggest that a lack of microbial stimulation in the first year of life can activate the genetic risk for asthma onset, while appropriate microbial exposure may prevent it. Furthermore, the presence of older children in the household has been found to facilitate the maturation of the gut microbiota and development of the immune system, potentially reducing the risk of asthma in the younger children, as confirmed in a subsequent study [56]. Another study established an association between the diversity and composition of the airway microbiota in asymptomatic infants and the subsequent risk of developing asthma by age six [57]. Specifically, certain bacterial taxa, such as *Veillonella*, *Prevotella*, and *Gemella*, have been associated with an increased risk of asthma. These bacteria were linked to an independent predictor of asthma—reduced TNF-α and IL-1β and increased CCL2 and CCL17 in airways. These results suggest that specific microbial taxa and immune interactions in newborns may predispose to asthma in childhood.

Some authors have demonstrated that the positive relationship between exclusive breastfeeding and the reduced risk of childhood asthma is mediated by the gut microbiome [58]. These findings add to the existing body of research [55,59,60,61] emphasizing the importance of the *Bifidobacterium* taxon in human breast milk and the early-life gut microbiome. Additionally, researchers have investigated the relationship between cesarean section delivery and childhood asthma risk, mediated by changes in gut microbiota [62]. Children whose gut microbiota composition remained with cesarean section microbes after one year had an increased risk of asthma. These findings indicate that appropriate maturation of the gut microbiota could moderate the increased asthma risk associated with cesarean section delivery.

Antibiotic administration during the first year of life and over the course of one’s lifetime significantly correlates with the incidence of asthma [63]. Exposure to antibiotics in early life has been found to significantly modify the gut microbiota, which mediated the correlation between antibiotic exposure in the first year of life and asthma diagnosis at age five [64].

Growing up on a farm has long been associated with a reduced risk of developing atopy [65,66,67,68]. Recent research indicates that this “farm effect”, which acts as a protection against asthma development, is mediated by the early-life gut microbiome, as evidenced by the reversal of airway inflammation in mice colonized with gut microbes depleted in children with asthma [61,69]. By modeling gut microbiome maturation between the age of 2–12 months, it was found that the estimated microbiome at 12 months of age highly correlated with previous farm exposure and a reduced risk of asthma in childhood [70]. Maturation and prediction of butyrate production was found to mediate the protective farm effect. 

Researchers examined the relationship between proximity to natural green spaces during infancy and the development of atopic sensitization at ages one and three years, while also investigating whether this relationship is mediated by gut microbiota composition at four months of age The study found that there was a protective effect of living near natural green spaces on the development of multiple inhalant atopic sensitizations at age three, which was mediated by *Actinobacteria* diversity changes in stool tests performed at four months. However, there was a positive correlation between nature proximity and sensitization to at least one food or inhaled allergen, which was not mediated by the gut microbiome. These results underscore the potential benefits of preserving natural urban green spaces to influence early-life gut microbiome development and long-term respiratory health outcomes, which is in line with previous findings [71,72] that also highlight the impact of urban-living factors on the gut microbiome and asthma [73,74]. Recent research showed that smoking may, on the other hand, change the lung microbiome in asthmatic individuals, and these changes could mediate and further contribute to the increased risk and severity of pulmonary disorders [75].

The effects of indoor dog exposure on the gut microbiome of infants was investigated, and it was found that dog-exposed infants had a more diverse gut microbiome with an accumulation of bacterial species, which was a statistically significant effect, especially in formula-fed children [76]. The enriched bacterial genera were *Fusobacterium*, *Collinsella*, *Ruminococcus*, *Clostridaceae*, and *Lachnospiraceae*. These results suggest that dog exposure alters the gut microbiome during infancy, potentially contributing to a decreased risk of allergies and asthma. Exposure to household furry pets has also been found to increase the infant gut microbiota abundance of the *Ruminococcus* and *Oscillospira* taxa, which have been negatively correlated with childhood atopy [77].

**Figure 3:**
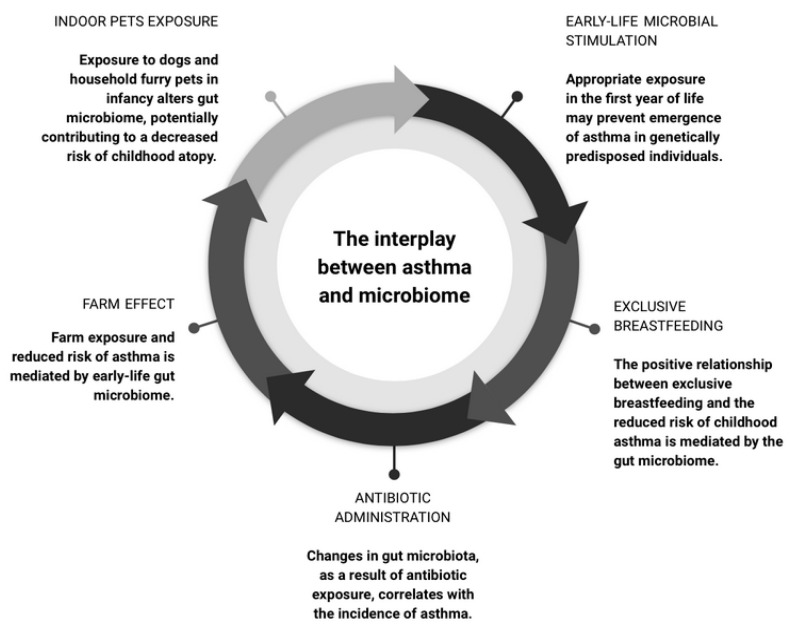
Summary of the interplay between asthma and the microbiome.

The interplay between asthma, obesity, and the microbiome is complex. Obese asthmatic patients are at risk of a severe disease course through increased inflammation and bronchial remodeling, which eventually contribute to a poor response to treatment [78]. Obesity exacerbates proinflammatory responses in asthma patients, and both asthma and obesity can alter the composition of the microbiome [74]. In individuals with both conditions, these effects appear to be additive. Notably, a negative correlation was observed between fecal *Akkermansia muciniphila* levels and asthma severity, and the administration of *A. muciniphila* has been found to significantly reduce airway hyperreactivity and inflammation in murine models, which shows promise in severe asthma treatment (Figure 3).

## 5. Treatment

Currently, the treatment for asthma is based on the use of inhaled corticosteroids (ICS). The 2020 Focused Updates to the Asthma Management Guidelines recommend the use of ICS as the preferred long-term control medication for all patients, regardless of the severity of the disease. For patients with persistent asthma, the preferred therapy is a low-dose ICS combined with a long-acting beta2-agonist (LABA). If asthma is still not well controlled, it is recommended to increase the dose of ICS or to add other medications, such as leukotriene modifiers or theophylline. It is vital to regularly monitor and adjust treatment based on asthma control and severity [79,80,81]. The pre-treatment bronchial microbiome of asthmatic patients was found to differ according to their responsiveness to corticosteroid treatment. Enrichment in *Hemophilus* correlated with a decreased response. The researchers hypothesized that this may be due to an enhancement of the bacterial xenobiotic degradation capacity [82]. Similar results were found in another study with severe asthma patients. The presence of Proteobacteria was linked with a TH17-related gene signature in airway epithelial tissue, worsening asthma control. What is more, Actinobacteria correlated with improving/stable asthma control and the bronchial epithelial gene expression of FKBP5, which is an indicator of steroid responsiveness [83].

Regarding the crucial role of the microbiome in asthma, alteration of the microbiota might act as a therapeutic or prophylactic strategy to enhance certain bacterial communities in the gut and lungs [84]. Therapeutic strategies aimed at modulating the microbiome in order to prevent or treat asthma include several different approaches: supplementation of prebiotics or probiotics, dietary interventions, and fecal microbiota transplantation.

Prebiotics are nondigestible oligosaccharides which selectively encourage the growth of beneficial bacteria, changing the constitution of the microbiota [85,86]. Fructo-oligosaccharides (FOS), galacto-oligosaccharides (GOS), and trans-galacto-oligosaccharides (TOS) represent the predominantly known groups of prebiotics [87]. Dietary products containing prebiotics are, e.g., onion, garlic, asparagus, wheat, oats and soybeans [88]. Currently, their production on a large scale has become prevalent due to their relatively low concentration in foods [89]. Prebiotics may alter the activity of the immune system, either directly or indirectly, via metabolites produced through fermentation or the probiotic-mediated modulation of gene expression [86]. Oligosaccharides of human breast milk are unique prebiotics. Studies have shown that they have direct effects on the development of the immune system of newborns by stimulating the production of interleukins and influencing the phenotype and proliferation of immune cells [90]. Cow’s milk, used for infant formula production, contains significantly lower amounts of oligosaccharides, which highlights the irreplaceable role of breastfeeding [91]. In adults, prebiotics increase phagocytosis, natural killer cell activity, and anti-inflammatory cytokine production [92]. Little is currently known about the use of prebiotics in asthma due to limited available data. A meta-analysis of two studies found no significant difference in infant asthma, although significant heterogeneity was found between studies [93]. On the other hand, a 2021 study found that children receiving prebiotics and synbiotics had lower asthma risk compared with the control groups and suggested the potential to reduce the incidence of asthma in children [94]. Furthermore, a meta-analysis from 2017 concluded that prebiotics decreased the risk of wheezing or asthma in comparison with the control group. However, this finding should be interpreted cautiously due to several limitations in the analyzed studies, such as the small number of patients or inconsistencies in defining wheezing and asthma [95]. Additional research is necessary to establish whether prebiotics can play a protective role in managing asthma.

**Figure 4:**
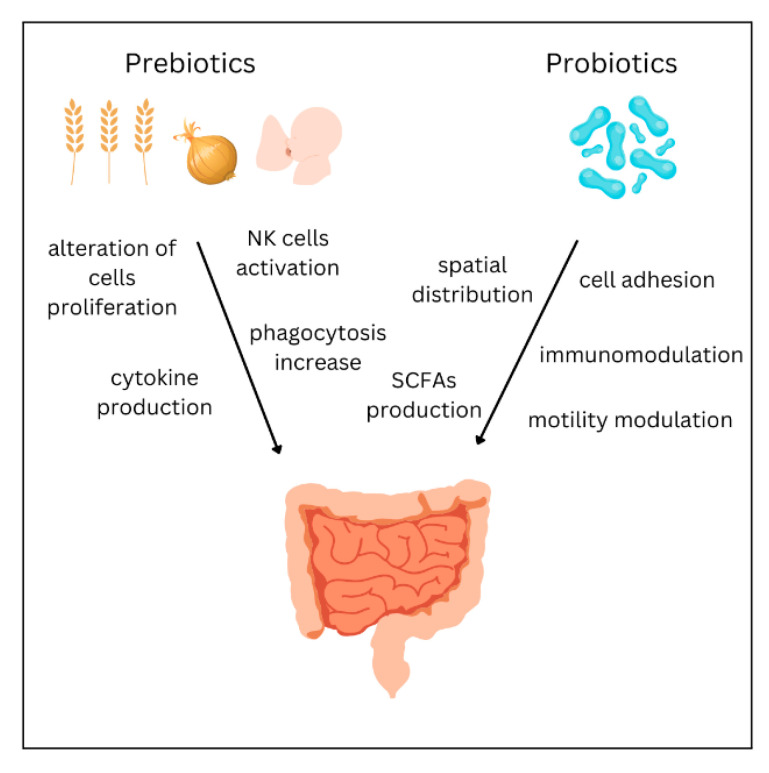
Mechanisms of action of prebiotics and probiotics.

Probiotics are live microorganisms administered to provide a health benefit when consumed in adequate amounts [96]. They are available in the form of dietary supplements or foods and comprise *Saccharomyces boulardii* yeast or lactic acid bacteria such as *Lactobacillus* and *Bifidobacterium* [97]. They can be found in both single-strain or multi-strain formulations. In some studies, probiotic mixtures appear to demonstrate higher effectiveness compared with single strains [98,99]. However, a recent 2021 meta-analysis concluded that their efficacy is similar [100]. Probiotics are used in treating antibiotic-associated diarrhea, gastrointestinal disorders, respiratory infections, bacterial vaginosis, and atopic eczema. Research efforts have been put into investigating the efficacy of probiotics in a number of various medical conditions, such as elevated cholesterol levels, cystic fibrosis hepatic encephalopathy, and diabetes [101,102]. The effects of probiotics are achievable through several different mechanisms that include cell adhesion, mucin production, pH modulation, and motility improvement [103]. Their efficacy is also connected with their spatial distribution in the gut lumen and the production of H_2_O_2_, SCFAs, and B group vitamins [104,105,106]. A very promising area is the anticancer impact of probiotics, which includes their influence on the immune system, cytokine production, and phagocytosis [107]. Numerous studies have examined the beneficial effect of probiotics, but the results are ambiguous due to differences in the strains and doses of probiotics used, the populations studied, and the study design [108]. Some studies have shown the prophylactic effect of probiotic supplementation [109,110]. However, the findings of most meta-analyses do not support it. The meta-analyses from 2013 and 2015 investigating the role of prenatal or early-life probiotic supplementation showed a reduction in the risk of atopic sensitization and eczema, but not a reduction in the risk of asthma or wheezing [95,111]. A 2020 meta-analysis showed similar results, apart from the subgroup of infants with atopic disease, in which the probiotics significantly reduced the incidence of wheezing [112]. A systematic review of probiotic supplementation in children with asthma found an association with fewer exacerbations, lower IL-4 levels, and higher interferon-γ levels. Nevertheless, there were no differences in asthma control tests, respiratory symptoms, or parameters of lung function [113]. Based on current knowledge, probiotic administration to reduce wheezing development is not recommended [114]. However, some most recent studies underline the efficacy of probiotics, especially highlighting the need to investigate specific probiotic strains. In a study by Ciprandi et al., the mixture of two strains (*Ligilactobacillus salivarius* LS01 and *Bifidobacterium breve* B632) significantly prevented wheezing and asthmatic exacerbations in children [115]. A study by Sangkanjanavanich et al. showed the effectiveness of *Bifidobacterium infantis* in improving lung function and control of uncontrolled asthma [116]. What is more, a 2022 meta-analysis indicates that probiotic intake improves quality of life and reduces symptoms of allergic asthma and allergic rhinitis [117]. Extended follow-up of ongoing trials, along with additional clinical and basic research, is necessary to determine the value of probiotics for asthma in children. Genetics, epigenetics, mechanisms of action, and personal responsiveness to specific strains should be the topic of interest in future studies [115]. The mechanisms of action of probiotics and prebiotics are summarized in Figure 4.

In Western countries, a diet with excess energy intake as well as the regular consumption of processed foods and limited consumption of fruit, vegetables, and whole grains is correlated with an increased asthma risk [118]. There is evidence that a diet rich in plant-based foods might be a protective factor against asthma development and improve symptoms by affecting the systemic inflammation, oxidation, and microbial composition as well as cytokine release and immune responses. [119]. For people with obesity, weight management may improve asthma outcomes [120]. A recent review found dietary factors to be important determinants of asthma risk in children, however it emphasized the need for further high-quality studies to allow recommendations [121]. A meta-analysis from 2022 found that prenatal vitamin D supplementation may be effective for the prevention of asthma [122]. There is conflicting evidence that the intake of omega-3 fatty acids during pregnancy and infancy may be associated with a reduced risk of childhood wheeze [123]. However, several limitations in the studies, such as the lack of standardized formats and doses, limit the ability to compare results and create recommendations [124]. Evidence on the supplementation of nutrients, such as vitamins C and E, selenium, magnesium, propolis extract or caffeine, is currently insufficient to establish an actual impact on asthma management [125].

Fecal microbiota transplantation (FMT) refers to the transfer of stool from a healthy donor into the colon of a patient with the goal of restoring the normal microbiota and gaining a therapeutic benefit [126]. Studies have not yet examined the efficacy of FMT in asthma. However, there is emerging interest in FMT for the management of extra-intestinal diseases such as metabolic, neuropsychiatric, autoimmune, and allergic disorders, in which the gut microbiota plays a role [127]. FMT may be more effective than probiotics due to the larger amounts of implemented bacteria as well as the ability to alter the microbiota for a longer term. The use of FMT seems promising in restoring immune homeostasis and improving asthma control, although considerable future research is needed [128].

## 6. Conclusions

The relationship between the microbiota and asthma is complex, with evidence suggesting that specific commensal microorganisms can play a significant role in the development of asthma. Antibiotic administration during the first year of life and over the course of one’s lifetime significantly correlates with the incidence of asthma, while the “farm effect”, proximity to natural green spaces during infancy, and exclusive breastfeeding have been associated with reduced asthma risk, a connection mediated by the early-life gut microbiome. The presence of older children in the household has been found to facilitate the maturation of the gut microbiota and development of the immune system, potentially reducing the risk of asthma in the younger children. Exposure to certain environmental microorganisms in early life may be necessary for the proper maturation of the immune system and prevention of asthma. Interactions between the microbiota and the host’s immune system strongly influence mechanisms that contribute to the development of asthma. Bacteria and their metabolites are responsible for molecular alterations in the host’s immune system; they can affect cell differentiation as well as modulate cell responses and the secretion of proinflammatory or protective factors, such as cytokines, interleukins, or antibodies. Microbial dysbiosis may lead to inflammatory system disturbances and contribute to the development of allergic diseases, especially asthma, by promoting opportunistic pathogen invasion caused by imbalanced immune responses. Considerable research effort has been put into determining the efficacy of various therapeutic strategies in asthma. Prebiotics, probiotics, and certain dietary strategies may have a prophylactic or therapeutic impact, but more high-quality studies are needed to draw clear conclusions.

## Figures and Tables

**Table 1:** The most common bacteria, fungi, and viruses in the human microbiome.

	Bacterial Genera/Phyla	Fungal Genera/Fungus Taxa	Virus Genera
**Gastrointestinal microbiome**	Bacteroides, Eubacterium, Peptococcus, Peptostreptococcus, Clostridium, Ruminococcus, Faecalibacterium, Escherichia, Lactobacillus	Candida, Saccharomyces, Aspergillus, Penicillium, Bullera, Pleospora, Rhodotorula, Trametes, Galactomyces	Bacteriophages
**Respiratory microbiome**	Prevotella, Streptococcus, Veillonella, Firmicutes, Bacteroidetes	Ascomycota, Basidiomycota, Candida, Saccharomyces, Penicillium, Cladosporium, Fusarium	Understudied

## Data Availability

Data sharing is not applicable as no datasets were generated or analyzed during the current study.
